# Perinatal programming of emotional brain circuits: an integrative view from systems to molecules

**DOI:** 10.3389/fnins.2014.00011

**Published:** 2014-02-05

**Authors:** Jörg Bock, Kathy Rether, Nicole Gröger, Lan Xie, Katharina Braun

**Affiliations:** ^1^PG “Epigenetics and Structural Plasticity”, Institute of Biology, Otto von Guericke University MagdeburgMagdeburg, Germany; ^2^Center for Behavioral Brain SciencesMagdeburg, Germany; ^3^Department of Zoology/Developmental Neurobiology, Institute of Biology, Otto von Guericke University MagdeburgMagdeburg, Germany

**Keywords:** early life stress, psychopathology, resilience, epigenetics, sex differences

## Abstract

Environmental influences such as perinatal stress have been shown to program the developing organism to adapt brain and behavioral functions to cope with daily life challenges. Evidence is now accumulating that the specific and individual effects of early life adversity on the functional development of brain and behavior emerge as a function of the type, intensity, timing and the duration of the adverse environment, and that early life stress (ELS) is a major risk factor for developing behavioral dysfunctions and mental disorders. Results from clinical as well as experimental studies in animal models support the hypothesis that ELS can induce functional “scars” in prefrontal and limbic brain areas, regions that are essential for emotional control, learning and memory functions. On the other hand, the concept of “stress inoculation” is emerging from more recent research, which revealed positive functional adaptations in response to ELS resulting in resilience against stress and other adversities later in life. Moreover, recent studies indicate that early life experiences and the resulting behavioral consequences can be transmitted to the next generation, leading to a transgenerational cycle of adverse or positive adaptations of brain function and behavior. In this review we propose a unifying view of stress vulnerability and resilience by connecting genetic predisposition and programming sensitivity to the context of experience-expectancy and transgenerational epigenetic traits. The adaptive maturation of stress responsive neural and endocrine systems requires environmental challenges to optimize their functions. Repeated environmental challenges can be viewed within the framework of the match/mismatch hypothesis, the outcome, psychopathology or resilience, depends on the respective predisposition and on the context later in life.

## The concept of experience-expectant brain development: windows of stress vulnerability and stress resilience—timing matters!

The neuroanatomist and Nobel price awardee Camillo Golgi stated in 1869 that “functional scars in the brain” might be the organic cause of mental disorders. A variety of studies have shown that during perinatal sensitive periods the environment exerts a critical impact on the maturation of brain structure and function (Weinstock, 2008; Korosi and Baram, 2009; Fox et al., 2010; Loman and Gunnar, 2010; Lucassen et al., 2013). Structural abnormalities related to early adverse experience are mostly found in brain regions that are involved in the control and mediation of emotionality, providing a direct link between childhood adversity and psychopathological behavior in adulthood (McCrory et al., 2010). Moreover, the outcome of stress exposure depends on the maturational status of a given brain region, e.g., disorders arising from exposure to adversity at times of frontal cortex development should differ from those of the hippocampus or the amygdala. The experience-*dependent* synaptic reorganization can be viewed as a general principle of perinatal brain development, where a genetic predisposition interacts with environmental and psychological “epigenetic” factors. As a consequence, synaptic circuitries adapt or maladapt to an adverse environment such as socio-emotional neglect, abuse and traumatic experience. This can result in dysfunctional neuronal systems, which might trigger the emergence of mental disorders later in life (Furukawa et al., 1999; Agid et al., 2000; Van Den Bergh et al., 2006; Cirulli et al., 2009).

In addition to the experience-dependent adaptation there is the often-overlooked concept of experience-*expectant* development, which was established by Greenough et al. (1987). According to this concept distinct developmental time periods exist, during which the brain expects and “waits” to interact with the environment, that is, only if the brain is exposed to a certain amount of experience its functions can be adapted and optimized. Joseph applied this concept to describe the environmental influences on neuronal development and its consequences for emotional development and attachment (Joseph, 1999). He postulated that “*deprived or abnormal rearing conditions induce severe disturbance in all aspects of social and emotional functioning, and affect the growth and survival of dendrites, axons, synapses, interneurons, neurons and glia.*” Moreover, he stated that “*immature limbic nuclei are experience-expectant and may be differentially injured depending on the age at which they suffer deprivation.*” Thus, if brain systems, which are relevant for emotionality, are deprived from adequate socio-emotional stimulation during critical developmental time windows, they may develop dysfunctional neuronal networks resulting in emotional retardation and pathological behavioral outcome.

Since a major hallmark of experience-dependent as well as experience-expectant development is the existence of developmental time windows, the behavioral outcome of perinatal adverse experience should be a function of the timing and duration of the stress exposure (Andersen, 2003; Andersen and Teicher, 2008). In a life cycle model of stress Lupien et al. (2009) eloquently outlined in which way exposure to stress during different stages in life can affect the developmental profiles of the amygdala, hippocampus and frontal cortex, areas that are involved in the regulation of the hypothalamus-pituitary-adrenal (HPA) axis. The authors stated “*from birth to 2 years of age the hippocampus is developing it might therefore be the brain area that is most vulnerable to the effects of stress at this time. By contrast, exposure to stress from birth to late childhood might lead to changes in amygdala volume, as this brain region continues to develop until the late 20s. During adolescence the hippocampus is fully organized, the amygdala is still developing and there is an important increase in frontal volume. Consequently, stress exposure during this period should have major effects on the frontal cortex*” (Lupien et al., 2009).

The mechanisms, which are critically involved in experience-expectant brain development are still poorly understood. Environmental factors can interact with genetically preprogrammed cellular events displaying distinct developmental timelines such as neurogenesis, cell migration and differentiation, formation and elimination of synapses and myelination (Rice and Barone, 2000). It is important to point out that different functional brain systems and brain regions and the different transmitter systems, including the modulatory transmitter systems dopamine, serotonin, acetylcholine and noradrenalin, as well as the excitatory glutamatergic and inhibitory GABA-ergic systems, follow different developmental timelines, with different trajectories in cortex, hippocampus and brainstem (Herlenius and Lagercrantz, 2004). Critical time windows have also been identified on the synaptic level for developing sensory and prefrontal cortical regions in the human brain (Huttenlocher, 1979; Huttenlocher and Dabholkar, 1997). Initially after birth sensory, motor and prefrontal regions undergo dramatic synaptic proliferation, which display specific developmental profiles for each cortical region. This increase or “overproduction” of synaptic connections is followed by “pruning” of synapses, which again shows specific temporal profiles for different cortical regions. This developmental synaptic selection process is most likely the neuronal substrate, which mediates the adaptation towards a given environment (Scheich, 1987; Scheich et al., 1991; Wolff and Missler, 1993), and which mediates volumetric differences of specific brain areas in patients after ELS exposure.

## The developing brain is a shifting target for adversity: the neurotoxicity and vulnerability hypotheses

As outlined in the previous chapter, critical or sensitive periods during mammalian brain development represent time windows of elevated synaptic plasticity, which show region- and neuron-specific timelines mediating vulnerability, but also may be “windows of opportunity” (Andersen, 2003) to establish resilience and improved stress coping. A recent study revealed that ELS during distinct developmental time windows was associated with volumetric reductions in the anterior cingulate cortex (ACC) and the insular cortex (Baker et al., 2013), areas that are essentially involved in the processing and control of emotional and cognitive processes and in the regulation of body homeostasis. This is in line with abnormalities of the prefrontal cortex in adults, which are associated with pediatric maltreatment-related posttraumatic stress disorder (PTSD) (De Bellis et al., 2002; Carrion et al., 2009), major depression induced by childhood stress (Frodl et al., 2010), harsh corporal punishment (Tomoda et al., 2009) and emotional maltreatment during childhood (Van Harmelen et al., 2010). More specifically, reductions in the volume of the orbitofrontal cortex, a prefrontal subregion involved in decision-making and the regulation of emotional and social behavior, have been found in individuals, who experienced physical abuse in childhood related to social difficulties (Hanson et al., 2010). Other brain regions affected by childhood maltreatment include the hippocampus and the amygdala (Woon and Hedges, 2008; Frodl et al., 2010; Teicher et al., 2012), key areas of the limbic system, which are involved in the regulation of stress responses particularly via the HPA-axis. The existence of region-specific sensitive time windows has been revealed by a study showing that childhood sexual abuse experienced between 3 and 5 years of age resulted in a reduction of hippocampal volume, whereas abuse between ages 14 and 16 years was associated with reduced gray matter volume in the frontal cortex (Andersen and Teicher, 2008).

However, from the clinical data the critical “hen and the egg” question remains unsolved: are these “functional scars” induced by adverse environmental conditions during critical stages of brain development, or should they rather be considered to be the cause for delayed, incomplete or inadequate neuronal development? As outlined in a recent review by Lupien et al. (2009) the *neurotoxicity hypothesis* (Sapolsky et al., 1986) claims that chronic glucocorticoid release (induced by extended stress exposure) increases stress-mediated neurodegeneration resulting in structural changes such as shrinkage or increase of a given brain region's volume. In contrast, the *vulnerability hypothesis* (Charney and Manji, 2004) proposes that differences in neuronal structure or the volume of a given brain region rather represent pre-existing risk factors to develop behavioral dysfunctions, which are induced by genetic predisposition and/or early stress exposure. The contribution of these mutually not exclusive perspectives remains to be further disentangled in systematic experimental approaches.

Moreover, the clinical observations raise another key question, whether “size matters.” Is the volumetric change of a given brain region the result of environmentally induced alterations in neuronal, dendritic and synaptic development, or is it the outcome of a genetically predisposed impaired development? And is a smaller volume of a brain region in fact an indicator of impaired function? Thus, a more detailed experimental and microscopic analysis is essential to assess in which way early life stress can modify the developmental trajectory of the brain on the synaptic level. Finally, the underlying developmental mechanisms as well as the transient or long-term impact of volumetric and structural changes in response to early adversity are not well understood. Stress, neglect or trauma may on one hand slow down or accelerate the speed of development of a given brain region during the time of exposure, whose functional maturation might eventually catch up and “recover” back to normal upon exposure to a stimulating, positive environment. On the other hand the developmental delay or retardation may result in permanent functional impairment, reflected by reduced brain volumes in adulthood. Is there a “time of no return,” can we “reopen” closed developmental time windows for therapy?

To address such questions on the cellular and molecular level, the systematic well-controlled experimental investigation of animal models for perinatal stress, trauma, and neglect, which mimic human early childhood trauma, neglect and abuse during different developmental time windows, is essential. Prenatal stress is a very early intervention during brain development, that is, exposure of pregnant dams to repeated stress situations during distinct gestational phases to stress the embryo in utero (Maccari et al., 2003; Weinstock, 2008; Charil et al., 2010; Pryce et al., 2011; Schroeder et al., 2013). The postnatal environment is a neonatal time window, during which the impact of a disturbance of mother/parent child-interactions and maternal care (acute and repeated maternal separation, paternal separation, parental separation, handling, maternal neglect) or natural variations in maternal care can be studied (for reviews see Meaney, 2001; Pryce et al., 2005; Sullivan et al., 2006; Rice et al., 2008; Cirulli et al., 2009; Oitzl et al., 2010; Bock and Braun, 2011; Schmidt, 2011; Lucassen et al., 2013). As a later developmental time window of stress exposure, post-weaning pre-puberty (juvenile) stress and its association to depressive-like behavior and anxiety can be investigated (Avital and Richter-Levin, 2005; Horovitz et al., 2012). Very recently, a novel animal model has been established, in which pregestational stress including the transgenerational effects on brain and behavioral development can be studied (Shachar-Dadon et al., 2009; Leshem and Schulkin, 2012; Zaidan et al., 2013).

## Early life stress affects neuronal and synaptic development and alters the excitation/inhibition balance of cortical neurons

Since the very first and most significant emotional early life experience for a newborn or child is embedded in the contact and relationship to its parents (Bowlby and King, 2004) it is reasonable to conclude that neonatal adverse environment such as emotional neglect, physical or sexual abuse and socio-emotional deprivation are risk factors for developing psychopathologies and mental disorders (Spitz, 1945; Skeels, 1966; Rutter, 1991; Draijer and Langeland, 1999; Furukawa et al., 1999; Agid et al., 2000; Beers and De Bellis, 2002; Van Den Bergh et al., 2006; Bale et al., 2010; Schury and Kolassa, 2012; Ehlert, 2013; see also Child Welfare Information Gateway, 2013). There is increasing evidence from experimental animal models that the behavioral dysfunctions and symptoms of mental illness induced by such perinatal traumatic experiences are associated with neurostructural and neurophysiologic alterations resulting in dysfunctional brain circuits (Rakic et al., 1994; Braun and Bogerts, 2001; Cirulli et al., 2009; Bale et al., 2010; Charil et al., 2010; McEwen, 2010; Bock and Braun, 2011; Weinstock, 2011; Baram et al., 2012; Maggio et al., 2012; Grigoryan and Segal, 2013). Particularly, the maturation of limbic brain regions and their connections to prefrontal cortical areas appear to be affected by early socio-emotional experiences. As outlined above these regions undergo experience-expectant synaptic selection processes in order to learn and optimize emotional behavioral responses. The very first emotional experience, which “programs” an individual's emotional development, is the establishment and maintenance of an emotional bond between a newborn and its mother or parents (Ainsworth, 1962; Bowlby and King, 2004). This neonatal learning event has been termed “filial imprinting” (Lorenz, 1935), and the underlying cellular mechanisms have been studied intensively in precocious avian species, such as the domestic chick. These classical animal models revealed that the first emotional experience induces synaptic reorganization in higher cognitive pallial regions, accompanied by specific metabolic, physiological, and neurochemical changes (Wallhäußer and Scheich, 1987; Bock et al., 1996, 1997; Bock and Braun, 1999; Horn, 2004).

On the other hand, disturbance of the emotional bond between caregiver and newborn, and even adverse experience of the mother during pregnancy show an equally strong effect on the development of prefrontal and limbic brain circuits in a region- and time-dependent manner. Prenatal stress appears to program the development of the offspring's hippocampal formation. Studies that were confined to the analysis of adult male offspring revealed a decrease in dendritic length and complexity in the CA1, CA3 and dentate gyrus after prenatal stress exposure (Hosseini-Sharifabad and Hadinedoushan, 2007; Martinez-Tellez et al., 2009). Similarly, it was reported that prenatal stress induces dendritic atrophy in CA3 pyramidal neurons in juvenile female rats (Jia et al., 2010) and reduces dendritic length and complexity of hippocampal neurons of 1-day-old rat offspring (Fujioka et al., 2006). A more comprehensive study in rats revealed that stress during the last gestational trimester induced a very distinct and sex-specific pattern of neuromorphologic changes in the hippocampal formation of prepubertal rats (Bock et al., 2011). Interestingly, some but not all of the stress-induced neuronal alterations could be reversed by neonatal handling of the offspring, an effect that was evident particularly in male offspring.

In addition to the stress-induced neuronal changes in the hippocampal formation, prenatal stress also induced significant sex-specific alterations in the anterior cingulate (ACC) and orbitofrontal (OFC) cortices. In these areas dendritic atrophy and decreased dendritic spine density was observed in layer III pyramidal neurons (Murmu et al., 2006). Dynamic alterations in dendritic spine density in the prefrontal cortex have also been reported after mild prenatal stress from embryonic days 12–16. Whereas this prenatal stress paradigm resulted in increased spine densities in the medial prefrontal cortex (mPFC) and the OFC at weaning age, a decrease in the mPFC and no effect in the OFC were observed in adult animals (Muhammad and Kolb, 2011; Mychasiuk et al., 2012).

On the structural level there is convincing evidence that early postnatal stress also programs the structural development of limbic and prefrontal cortical brain areas. In mice, repeated 3 h/day maternal separation induced significant increases of dendritic complexity and dendritic spine number in the hippocampal CA3 (Xie et al., 2013). Furthermore, experiments in rats revealed that the structural outcome of ELS exposure is related to the maturational state of a given brain region. ELS (maternal separation between postnatal days 2 and 20) exerts a negative effect on synaptic density in the hippocampus of rats, whereas later stress exposure (social stress between postnatal days 30 and 35) affects synaptic density in the prefrontal cortex (Andersen and Teicher, 2004, 2008).

Rodent models are also ideal tools to investigate the contribution of HPA function since in rodents the HPA-axis shows a distinct developmental profile around birth. In rats (and other rodents) the neonatal period is characterized by very low basal levels of corticosterone and a relative non-responsiveness to mild external stressors (Rosenfeld et al., 1992; Levine, 2001). This so-called stress hyporesponsive period (SHRP) of the HPA-axis was proposed to protect the developing juvenile brain from the deteriorating effects of high levels of stress hormones (De Kloet et al., 1988; Meaney et al., 1991). Using brief episodes of ELS (maternal separation) it was demonstrated that the extent and the direction of stress-induced neuromorphologic changes are strongly correlated to the SHRP. While maternal separation prior to the SHRP from postnatal day 1–3 decreased the density of dendritic spine synapses in the ACC, maternal separation from postnatal day 14–16 increased dendritic spine densities (Bock et al., 2005). Maternal separation during the SHRP had no effect on dendritic spine densities. Interestingly, this profile was specific for pyramidal neurons located in layer II/III. In contrast, layer V pyramidal neurons underwent a reduction of spine density only in those pups, which were stressed during the SHRP (Gos et al., 2008).

A later critical time window of development is weaning, when under laboratory conditions young rat pups are permanently removed from their mother. This major (stressful) event in the life of a young animal has a significant impact on prefrontal neuronal development (Ferdman et al., 2007; Bock et al., 2008). Moreover, it has been shown that differences in maternal care critically influence hippocampal synaptogenesis associated with cognitive development in rats (Liu et al., 2000). An elegant model of fragmented maternal care has been established for rodents, in which maternal neglect and early life psychosocial stress are induced by reducing bedding and nesting materials in the home cage (Baram et al., 2012). This model has been shown to induce cognitive and emotional dysfunctions and the observed learning deficits were associated with a reduction of synapses and dendritic spines and dendritic atrophy in the hippocampus (Brunson et al., 2005).

**Figure 1:**
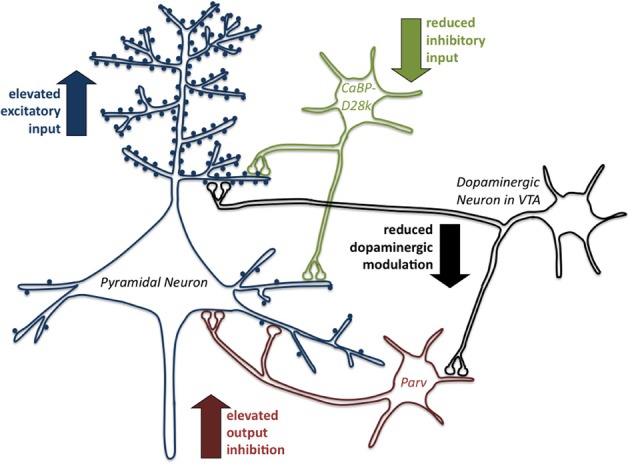
**Early life stress induces inhibitory/excitatory dysbalance in the prefrontal anterior cingulate cortex**. The scheme summarizes the changes of excitatory and inhibitory systems in the anterior cingulate cortex (ACC) induced by early life stress (repeated parental separation) in degus (*Octodon degus*). At first the excitatory input on pyramidal neurons is enhanced in response to early life stress since enhanced density of dendritic spines is observed, an effect indicating a disturbance in selective synaptic pruning. This effect is amplified by a reduction of inhibitory input reflected by reduction in CaBP-positive GABAergic interneurons. The resulting dysbalance in the system also becomes apparent in an elevated output inhibition by an increase in Parv-positive GABAergic interneurons. In addition the modulatory function of dopamine on the system is reduced. Overall, the observed alterations indicate a dysbalance of the excitatory and inhibitory modulation of pyramidal neuron activity in the prefrontal anterior cingulate cortex as a result of early life stress experience, which presumably underlies the described behavioral dysfunctions (for details see text).

Detailed and comprehensive information about the influence of early traumatic experiences on excitatory and inhibitory systems that modulate signal processing in cortical neurons comes from a series of experiments (Figure 1) in a precocious rodent, the degu (trumpet-tailed rat, *Octodon degus*). The degu has become an established animal model to study the development of social behavior and emotional experience during postnatal and adolescent development (Colonnello et al., 2011) and to analyze the impact of early life stress on the development of prefronto-limbic brain circuits (Bock and Braun, 2011; Braun and Bock, 2011). This precocious, diurnal South American rodent lives in complex social family structures, families are biparental and degu pups have been shown to develop a strong attachment to both parents (Fuchs et al., 2010). In contrast to the classical laboratory rodents mice and rats, degus are born with relatively mature sensory systems (open ears and open eyes) and thus can perceive and interact with their social environment in a much more elaborate way. It has been shown that a 1 h period of parental separation in these animals leads to a strong increase in stress hormone levels (Gruss et al., 2006). As revealed by a functional imaging study, this stress experience was accompanied by a dramatic decrease in brain activity in a number of brain areas, including areas of the limbic system and particularly the prefrontal cortex (Bock et al., 2012). With respect to neuronal development, that is very likely to be directly influenced by the reduced brain activity during separation, it was shown that animals exposed to repeated parental separation during the first three weeks of life end up with higher dendritic spine densities in the prefrontal ACC and in the infralimbic cortex when compared to unstressed controls (Helmeke et al., 2001; Ovtscharoff and Braun, 2001). This finding is most likely the result of delayed or permanently impaired synaptic pruning during prefrontal cortical development. Stress-induced changes in dendritic spine density were also found in the amygdala, where the stressed degus ended up with reduced levels and in the hippocampus where stress-induced elevated spine densities are found in the CA1 and reduced spine densities in the dentate gyrus (Poeggel et al., 2003). Besides these changes of excitatory spine synapses in prefronto-limbic networks, there is evidence that inhibitory systems appear to be changed in parallel as response to early life stress overall inducing a dysbalance of synaptic input and neuronal output in the affected networks. For example, the stress-induced increases in excitatory spine density in the prefrontal cortex are accompanied by a decrease of presumably inhibitory shaft synapses on the same neurons (Ovtscharoff and Braun, 2001). Moreover, the described synaptic alterations are paralleled by changes of inhibitory GABAergic interneurons (Helmeke et al., 2008; Seidel et al., 2008). In addition to excitatory and inhibitory systems, monoaminergic pathways appear to be particularly vulnerable to stress exposure during early childhood. Stressed degu pups, display specific alterations of dopaminergic and serotonergic fiber innervation in the prefrontal cortex, including the ACC, infralimbic cortex, prelimbic cortex and orbitofrontal cortex, in the hippocampal formation and in the nucleus accumbens (Braun et al., 2000; Gos et al., 2006; Kunzler et al., 2013). For the nucleus accumbens a recent study also described an increase in dopamine transporter density, which could be observed in adult degus that had been exposed to parental separation during childhood (Kunzler et al., 2013). As summarized in Figure 1, particularly the changes in the excitatory and inhibitory systems in the prefrontal ACC indicate a dysbalance in small neuronal feedback loops that regulate the activity of the pyramidal neurons and provide a substrate for the development of dysfunctional large-scale neuronal networks (see below) that underlie the behavioral deficits observed after early life stress. Indeed, stressed degu pups develop hyperactive behavior and a disturbed responsiveness toward species-specific vocalizations such as the voice of the own mother (Braun et al., 2003).

## Epigenetic changes induced by early life stress (re)program brain structural, neurochemical and behavioral development

Epigenetic mechanisms are considered the interface between early environmental influences and genetically programmed developmental processes in the brain, including the maturation of neuronal dendrites and synaptic connectivity. Thus, it appears likely that the experience- and ELS-induced neuronal and synaptic changes are the result of epigenetic changes in the brain. Epigenetic processes are most commonly defined as the ensemble of alterations in gene functions that are heritable through both mitosis and meiosis, but that cannot be explained by changes in the DNA sequence itself (Levenson and Sweatt, 2005; Graeff and Mansuy, 2008). At the molecular level, epigenetic mechanisms are biochemical modifications of the DNA and histone proteins, the major constituents of chromatin. They include direct modifications of the DNA, through DNA methylation at CpG islands and very specific modifications of histone proteins such as acetylation, phosphorylation and methylation. Depending on the type of modification this can result in actively transcribed or silenced genes (Graeff and Mansuy, 2008; Sananbenesi and Fischer, 2009). Such epigenetic alterations mediate the relationship between early life experiences including childhood maltreatment and the long-term behavioral and most likely also the neuronal outcome of these experiences (for reviews see Mueller and Bale, 2008; Champagne and Curley, 2009; Fagiolini et al., 2009; Hoffmann and Spengler, 2012; Kundakovic et al., 2013; Lutz and Turecki, 2013; Szyf, 2013). The first evidence for epigenetic alterations associated with maternal care came from a study using the classical animal model of naturally occurring variations of maternal care (high licking/grooming vs. low licking/grooming) in rats (Liu et al., 1997; Meaney, 2001). These variations in maternal care have strong effects on endocrine and behavioral responses. For example, offspring from high licking/grooming mothers show reduced hypothalamic-pituitary-adrenal (HPA) axis reactivity to stressful experiences, decreased anxiety-like behaviors and improved learning capacities in adulthood. In a series of experiments it could be shown that offspring of high licking/grooming dams show increased expression of hippocampal glucocorticoid receptors (GR) related to a decreased reactivity to stress, because of a strengthened negative feedback onto the HPA-axis. Interestingly, these effects were correlated to decreased methylation levels at the neuron-specific exon 1_7_ GR promoter (Weaver et al., 2004). In parallel, histone acetylation is facilitated, which together with the changes in DNA methylation results in an increased GR transcription (Weaver et al., 2004). Evidence for epigenetically mediated programming effects of early adverse experiences came from a study applying daily 3 h maternal separation during postnatal days 1–10 to induce early life stress in mice. In this study a persistent increase of arginine vasopressin (AVP) in the hypothalamus was associated with a sustained DNA hypomethylation at DNA-binding sites for MeCP2 that regulate activity-dependent transcription of the Avp gene (Murgatroyd et al., 2009). Also, there is evidence for a lasting epigenetic influence of early life adversity on the BDNF gene. In this approach infant rats were exposed during the first postnatal week to dams that displayed abusive behavior. As adults the maltreated rats displayed persisting changes in the methylation of BDNF DNA causing alterations in BDNF gene expression (Roth et al., 2009). The effect of parental care on the epigenetic regulation of hippocampal GR expression in humans has been revealed in a study investigating postmortem hippocampal tissue. In this pioneering study the authors were able to show that suicide victims with a history of childhood abuse displayed decreased levels of neuron-specific GR mRNA associated with increased DNA methylation in the promoter region of this gene (McGowan et al., 2009). Interestingly, some studies revealed that the epigenetic marks induced by environmental effects might be transmitted across several generations (Bohacek et al., 2013). For example, mice that were exposed to chronic and unpredictable maternal separation until postnatal day 14 develop depressive-like behaviors and deficits in coping with stress in adulthood (Franklin et al., 2010). Most of the behavioral alterations could also be found in the offspring of male animals with early life stress history, indicating a transgenerational transmission. This assumption was supported by the finding that maternal separation induced specific changes of DNA methylation patterns in the germline of the separated animals. Interestingly, comparable changes of DNA methylation were also found in the brains of the separated animals' offspring (Franklin et al., 2010). However, besides this germ line dependent transgenerational transmission of environmental effects, behavioral alterations can also be transmitted across generations via behavioral or social transmission. This behavioral/social transmission is germline independent. That means a certain maternal behavior (supportive or abusive) has to be reinstated at each generation by mother-child interactions (Weaver et al., 2004; Champagne, 2008).

So far, most studies revealed evidence for stable/persistent epigenetic marks. However, it has to be pointed out here that the establishment of permanent epigenetic marks is a multistep process starting with immediate epigenetic alterations as a direct response to early life experience, most of these rapid changes are dynamic and transient (Meaney and Ferguson-Smith, 2010; Dudley et al., 2011). Such transient and dynamic epigenetic changes in the brain have been described for the expression of histone deacetylases (HDACs) and specific acetylations of histone H4 in mice after repeated 3h maternal separation during the first two weeks of life (Levine et al., 2012). In a recent study we tested the hypothesis that early life stress (maternal separation) induces rapid alterations in the acetylation of H3 and H4. Indeed, we were able to show that repeated periods of maternal separation during early childhood (3 h per day from postnatal day 14 to 16) induce a rapid increase in the acetylation of H3 and H4 in the hippocampus, which is measurable as fast as 30 min after the last separation period (Xie et al., 2013). Moreover, our results revealed a direct correlation between the elevated histone acetylation and an increase in the expression of the synaptic plasticity genes arc and egr1 (Xie et al., 2013).

## Programming of large-scale neuronal networks by early life experience: connectivity matters!

With respect to the processing and execution of social and emotional behaviors, and also for complex learning and higher cognitive competence it has to be emphasized that these functions are not restricted to the function of a single brain region, but are mediated by complex, orchestrated and fine-tuned interactions of large-scale neuronal networks (McIntosh and Gonzalez-Lima, 1998; Nair and Gonzalez-Lima, 1999; Nair et al., 2001). The basic question is, how do early life experiences affect or “program” the maturation of functional neuronal circuits?

It is hypothesized that a well balanced and coordinated interregional neuronal activity is essential for the activity dependent maturation of neuronal networks, and that disturbances in these networks may be associated with the etiology of neurodevelopmental disorders (Uhlhaas et al., 2010; Stam and Van Straaten, 2012) and various brain based disorders such as depression, obsessive-compulsive disorder, autism, schizophrenia and Alzheimer's disease (Bassett and Bullmore, 2009; Minshew and Keller, 2010; Del Casale et al., 2011; Hulvershorn et al., 2011; Liston et al., 2011). Along this line, functional imaging in awake, freely moving rodents (*Octodon degus*) support the concept that early life stress alters not only the activity of distinct brain regions but also the interregional activity patterns. It was demonstrated that during acute stress (separation from the parents) brain activity was down regulated in distinct prefrontal and limbic areas, and in addition altered interregional functional coupling among these regions (Bock et al., 2012). The high level of positive interregional correlations particularly between different subregions of the prefrontal cortex and limbic brain areas seen under unstressed control conditions was dramatically decreased during acute stress exposure. In particular, the OFC and the cingulate cortex, areas that are strongly related to executive function and decision-making, are almost completely uncoupled from the other brain regions. This indicates that the functional coupling within these circuits becomes increasingly disturbed.

The question arises whether and in which way such stress induced acute changes in functional coupling during early childhood may become chronic or lead to long-term stable alterations of network connectivity that are presumably associated with psychopathological behavior later in life. Evidence in support of this view arises from studies, which demonstrated that the interregional connectivity appears to be disturbed in response to childhood adverse experience, reflected by reductions in different areas of the corpus callosum (De Bellis et al., 2002; Teicher et al., 2004). A recent study focused on the influence of childhood maltreatment on the development of the network architecture within a number of cortical regions. Magnetic resonance imaging in 18–25 year old males and females with a history of childhood maltreatment revealed that maltreatment was associated with decreased centrality in a number of cortical areas (Teicher et al., 2013). This indicates the importance of interregional connectivity and correlated brain activity, in particular for regions involved in emotional regulation, aspects of theory of mind and enhanced centrality in brain areas involved in internal emotional processing and self-awareness (Teicher et al., 2013).

## Stress inoculation, resilience and the match/mismatch hypothesis of early life stress

The dynamic processes promoting the maintenance of mental health in the face of severe adversity or trauma are called resilience (Cicchetti, 2010; Herrman et al., 2011). Thus, the term resilience describes an individual's ability to keep a stable efficient maintenance of allostasis throughout development although it has been exposed to multiple stressors throughout its life (McEwen, 1998; Feder et al., 2009). Several factors influence an individual's path towards maladaptation/psychopathology or resilience: the biological constitution as well as the psychological organization, current experiences, characteristics of the adverse events and the social context. Knowledge of the underlying dynamic interactions between risk and protective factors and their progress during a child's development are essential for the design of prevention models. So far, most studies on resilience after early life stress have focused on behavioral and psychosocial factors, but the simultaneous investigation of psychological as well as biological protective factors is essential for a deeper understanding of the pathway leading to vulnerability or resilience (Curtis and Cicchetti, 2003; Charney, 2004; Masten, 2007). Despite the interest in resilience, it has been difficult to validate this construct in humans. This is, in part, due to our lack of knowledge of certain interrelations: which psychological constructs associated with resilience are altered by stress exposure during critical developmental time windows? Are resilient individuals born or made?

There is accumulating evidence from clinical and animal studies that stress experience early in life can promote adaptive effects on emotional and cognitive development, resulting in resilience to stressful experiences encountered later in life. These observations raised a “stress inoculation-induced resilience” hypothesis which is supported by a number of studies in humans, non-human primates and rodents (Boyce and Ellis, 2005; Lyons and Parker, 2007; Feder et al., 2009; Gunnar et al., 2009; Katz et al., 2009; Oitzl et al., 2010; Dudley et al., 2011; Macri et al., 2011; Parker and Maestripieri, 2011; Daskalakis et al., 2013; Karatsoreos and McEwen, 2013). Clinical studies provide evidence that stress during childhood correlates with diminished increases in salivary cortisol responses to the Trier Social Stress Test (Gunnar et al., 2009), lower cerebrospinal fluid (CSF) levels of corticotropin-releasing-factor (CRF) in healthy adults (Carpenter et al., 2004), and diminished cardiovascular responses during stressful laboratory tests (Boyce and Chesterman, 1990). Similarly, it has been shown that both, women and men with childhood stress experience who successfully coped with it, as adults display a better coping with stressful experiences (Forest, 1990; Khoshaba and Maddi, 1999). A specific stress inoculation paradigm has been established in non-human primates to produce resilience. In this model, squirrel monkeys are exposed to a 1h weekly separation between 17 and 27 weeks of age, a critical developmental period during which young monkeys become nutritionally independent (Parker et al., 2004). These stress “inoculated” monkeys display reduced HPA-activity and reduced anxiety in response to stressors later in life, due to better self-regulation of emotional arousal (Parker et al., 2004). Timing of stress exposure is one of the critical parameters mediating stress resilience. In the primate study relatively long intervals for recovery between the stress episodes were provided, giving the animals repeated opportunities to regain emotional balance. In addition, stress exposure was induced at a time when the juvenile monkey started to become independent, thus the stressor may not have emotionally overwhelmed the animals and induced “helpless” behavior, but may rather have trained them to acquire active coping strategies.

Studies in laboratory rodents also provide support for the stress inoculation hypothesis and the programming of resilience towards stress. One of the first experimental evidence comes from studies in the mid 50s, where it was shown in albino rats that a specific type of repeated handling, termed “gentling,” that started immediately after weaning, reduced physiological damage to specific organs such as the heart and reduced fearfulness in adult animals (Weininger, 1954). Levine and collaborators then demonstrated that brief periods of maternal separation during the first three postnatal weeks, which they termed ”handling,” induced a reduction of stress-mediated effects later in life (Levine, 1957, 1960; Levine et al., 1957). The neonatal “handling” paradigm was applied in a series of studies, which aimed to identify and characterize the behavioral, endocrine, neurochemical and structural correlates of stress resilience. There is now convincing evidence derived from the seminal work of Meaney et al., who demonstrated that the effects of neonatal handling are mediated via intensification of maternal care (Meaney, 2001). Enhanced sensory stimulation through higher rates of licking and grooming by the dams induced by neonatal handling of the pups induces physiological responses related to a reduced fearfulness, improved emotional and reduced adrenocortical reactivity and behavioral stress responsiveness (Meaney et al., 1988; Liu et al., 1997). This is in line with the observation that naturally occurring variability in maternal care has similar programming effects on stress responses in the offspring (Liu et al., 1997). Interestingly, the quality of maternal care can be transmitted across generations in a non-genomic way (Francis et al., 1999).

The ELS-induced changes in emotionality and the improvement of stress coping may at least in part contribute to the cognitive improvement, which has been reported as a consequence of early emotional experience (Levine, 1956; Weiner et al., 1985; Zaharia et al., 1996; Lehmann et al., 1999; Steimer and Driscoll, 2003), and thereby further support the stress inoculation hypothesis. Additional support for this hypothesis is provided by our studies in degus (*Octodon degus*) and laboratory rats using an aversive learning paradigm. Early life stress (daily separation from the parents and siblings during the first three weeks of life) results in improved performance in a two-way active avoidance paradigm in both, male and female offspring (Schäble et al., 2007; Abraham and Gruss, 2010). These findings indicate that repeated early life stress exposure might result in more active and efficient coping with stressful situations and can also be discussed in the context of the match/mismatch stress hypothesis (Oitzl et al., 2010; Schmidt, 2011; Frankenhuis and Del Giudice, 2012; Nederhof and Schmidt, 2012). This hypothesis claims that the outcome of early life stress exposure is not necessarily pathological, but can also trigger adaptive processes and is explained with the concept of a predictive adaptive response, that is, stress experience and stress coping strategies acquired in the past (e.g., childhood or adolescence) can be “applied” to cope with future environmental conditions in an adaptive way (match) (Gluckman et al., 2005). Consequently, a greater risk for a pathological outcome is predicted for a mismatch between the environmental conditions experienced during early phases of development and the environment experienced (and expected) later in life (Gluckman et al., 2007). Support for this hypothesis arises from a recent study, which showed that early life stress (repeated maternal separation) in heterozygous serotonin transporter knockout rats and wild type controls improved adult stress coping behavior (Van Der Doelen et al., 2013). Along the same line it was shown that the outcome of early adversity or neglect is not necessarily detrimental (Champagne, 2008). Offspring of neglecting dams displayed lower cognitive performance under basal (non-stress) conditions compared to offspring of supportive dams (mismatch). However, within a stressful situation, which is comparable to their early-life experience, offspring of neglecting mothers performed better compared to the offspring of supportive dams (match).

However, even though growing up in a continuously “matching” environment with repeated exposure to the same stressors may help the individual to develop a successful coping strategy, which he/she can use throughout life, the price for this potential behavioral advantage may lie in a reduced behavioral flexibility. In other words, once an individual learns a specific behavioral strategy during childhood it may be “stuck” with it for the rest of his/her life. In line with this view are observations in young mice using an aversive learning paradigm (Spröwitz et al., 2013). This study revealed that infant mice, which learned to escape from a footshock, maintain this escape strategy until adulthood, instead of switching to a more efficient avoidance strategy.

In contrast, being challenged once in a while by “mismatching” environments during childhood and adolescence may stimulate the brain to adapt and to modify and optimize a behavioral strategy according to a novel situation. Thus, exposure to mismatch may on one hand encourage behavioral flexibility, and on the other hand it may be applied as a strategy for therapeutic intervention to overcome behavioral rigidity.

## Vulnerability and resilience towards early life stress: sex matters!

Although early adverse experience can be a risk factor for the development of psychopathological behavior later in life for both, women and men, there is a considerable sex-bias in the prevalence of early adversity-induced disorders. However, the literature in this topic is still quite controversial and does not yet allow compiling a coherent picture of sex-specific stress vulnerability or resilience. For example, females experiencing trauma, physical abuse or maternal distress during infancy have been described to show higher rates of depression, anxiety and PTSD compared to males (Baker and Shalhoub-Kevorkian, 1999; Macmillan et al., 2001; Pitzer et al., 2011). In contrast, males appear more vulnerable to developing schizophrenic symptoms in response to perinatal stress (Van Os and Selten, 1998). Associated with prenatal stress, there is evidence that boys suffer from behavioral problems earlier in development (at one year of age; Gerardin et al., 2011), while girls display stronger effects during later life periods (Buss et al., 2011, 2012). Such sex-specific stress-induced behavioral differences are accompanied by differences in specific brain structures such as the amygdala, where girls show an increased volume compared to boys (Buss et al., 2012). Furthermore, it has been revealed that birth weight predicts hippocampal volume in adulthood in female subjects reporting low maternal care but not in males, indicating that an adverse postnatal environment modulates neurodevelopmental consequences of prenatal risk in a sex-specific manner (Buss et al., 2007).

On the neuroendocrine level, there is evidence that gender is a significant modulator of the relationship between childhood adversity and HPA-axis activity. In a recent study it has been shown that exposure to early trauma is positively associated with baseline corticotropin in women, whereas there was a negative association in men. In contrast, severe trauma was strongly positively associated with corticotropin response to CRH challenge in men but not in women (DeSantis et al., 2011). Furthermore, programming effects of pre- and postnatal maternal mood on sympathetic nervous system reactivity in response to physiological stressors appear to be restricted to males (Vedhara et al., 2012).

However, most of what is known about the effects of early life stress on the maturation of brain function arises from studies on male individuals, especially in experimental animal models, which clearly illustrates the need for a greater emphasis on sex differences in neuroscience research (Beery and Zucker, 2011). Consequently, there are a growing number of animal studies, which intend to deepen knowledge about sex-specific effects of early adversity on the behavioral, structural, physiological as well as on the molecular level.

On the behavioral level, concerning emotional and cognitive aspects, sex-specific effects were frequently reported in rodent studies on prenatal as well as postnatal stress. However, sex-bias is not consistent in terms of the direction of the behavioral outcome. For example, a number of studies in rats and mice report different emotional traits to be predominantly affected in males by diverse pre- and postnatal paradigms (Wigger and Neumann, 1999; Barna et al., 2003; Mueller and Bale, 2008; Franklin et al., 2010; Freund et al., 2013; Kundakovic et al., 2013). In contrast, other studies report opposite findings, such as increased anxiety and depressive-like behavior predominantly in prenatally stressed females, depicting parallels to human studies (Bowman et al., 2004; Zagron and Weinstock, 2006; Behan et al., 2011; Schroeder et al., 2013). Similarly, perinatal stress exposure has been shown to affect cognitive abilities such as spatial learning predominantly in male animals (Bowman et al., 2004; Zagron and Weinstock, 2006; Salomon et al., 2011), while other cognitive abilities such as object recognition and passive avoidance learning are reported to be more affected in females (Gue et al., 2004; Marco et al., 2013).

Matching investigations in humans, rodent studies revealed that males are more affected by hyperactivity and increased risk taking after early stress experience (Kundakovic et al., 2013; Schroeder et al., 2013). Specifically for prenatal stress, there is evidence that the time window of stress exposure during gestation is associated with sex-specific behavioral outcomes. While male animals appear to be more affected when exposed to stress early in gestation, behavioral changes in females are more pronounced when stress appeared during later gestational periods (Li et al., 2008; Mueller and Bale, 2008). Similar findings were described in humans (De Bruijn et al., 2009). In contrast to the so far cited studies, there is increasing evidence for positive outcomes of early childhood adversity, which occur especially in females, indicated by reduced anxiety and increased cognitive abilities (McIntosh et al., 1999; Zuena et al., 2008; Biala et al., 2011; Leon Rodriguez and Duenas, 2013).

**Figure 2:**
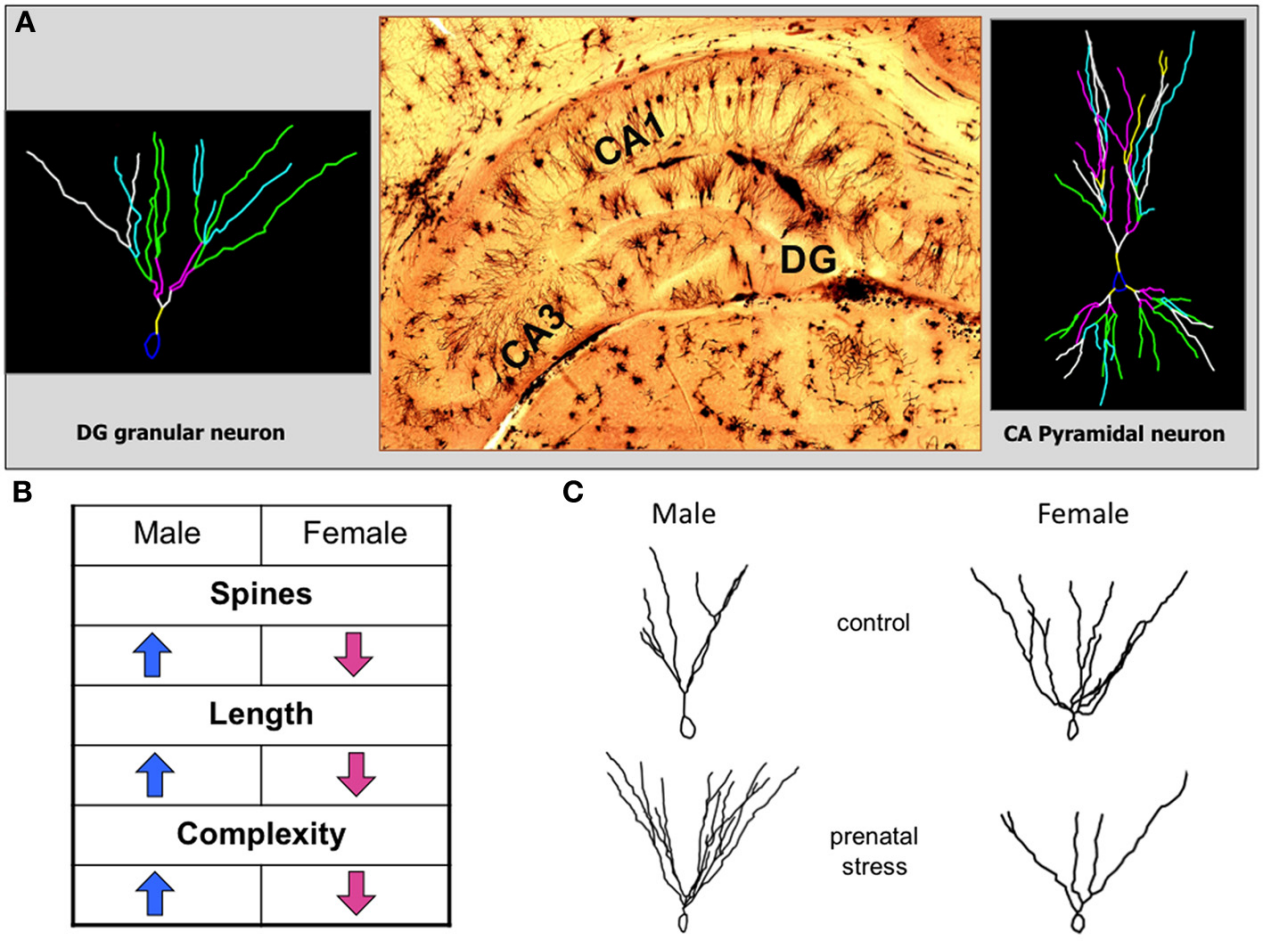
**Opposing effects of prenatal stress exposure on dentate gyrus granular neurons in male and female offspring. (A)** The histological picture in the middle shows a representative image of the hippocampal formation stained with the classical Golgi-Cox technique. On the left a representative reconstruction of a dentate gyrus granular neuron is shown, on the right a representative reconstruction of a CA pyramidal neuron. **(B)** The table indicates the significant changes of neuromorphologic parameters of dentate gyrus granular neuron dendrites after prenatal stress. Male and female offspring are affected in opposite directions. **(C)** Representative reconstructions of dentate gyrus granular neurons in male and female offspring of control and prenatally stressed dams showing the opposing sex-specific alterations of dendritic length and complexity.

With regard to structural neuronal and synaptic consequences in the brain after exposure to early life adversity, a variety of studies on prenatal stress, predominantly in rats (Murmu et al., 2006; Mandyam et al., 2008; Zuena et al., 2008; Bock et al., 2011) but also in mice (Behan et al., 2011) report region-specific differences between male and female animals. For example, stress applied during the last trimester of gestation in rats, induced an increase in dendritic length and complexity in the hippocampal denate gyrus and a decrease of the same parameters in the prefrontal cortex, an effect that was restricted to male offspring (Murmu et al., 2006; Bock et al., 2011). Moreover, dendritic length, complexity and spine density in the dentate gyrus were changed in opposite directions in male and female offspring (Figure 2). While the male offspring of rats stressed during gestation ended up with larger and more complex dendrites and higher spine densities, their sisters ended up with shrunken dendrites and lower spine density (Bock et al., 2011).

There is also evidence for a sex-specific influence of prenatal stress on hippocampal neurogenesis, as indicated by increased cell death (Mandyam et al., 2008) and reduced survival of newborn cells (Zuena et al., 2008), which was predominantly observed in male animals. Also, abnormal ultrastructural appearance of hippocampal neurons and myelin sheaths, and more degenerating neurons we reported to be more pronounced in males following prenatal stress (Xu et al., 2013).

In addition to stress-induced changes of neuronal morphology, sex-specific glial changes were found after prenatal stress, e.g., only female but not male offspring showed a reduction in hippocampal glial count (Behan et al., 2011).

Sex-specific effects of perinatal stress exposure have also been reported for the hypothalamus, which exerts control on endocrine systems to maintain homeostasis, including physiological mechanisms after stress experiences. For example, Reznikov and coworkers found sexually dimorphic changes in neuronal cell nuclei volumes in the suprachiasmatic nucleus of 10-day-old pups after prenatal stress (Reznikov et al., 1999). In the fetal PVN prenatal stress-induced apoptosis appears to be more pronounced in females than in males (Tobe et al., 2005). Also, it has been shown that different parameters in the hypothalamus such as cell death and proliferation as well as astrocyte and synaptic markers, respond differentially in males and females following both, prenatal and adult stress exposure (Garcia-Caceres et al., 2010). There is also evidence for sex-specific changes in neurochemical profiles regarding dopaminergic, serotonergic and GABAergic systems in rats and degus in response to early stress (Reznikov et al., 1999; Ziabreva et al., 2003; Jezierski et al., 2006; Barbosa Neto et al., 2012; Leon Rodriguez and Duenas, 2013).

Recently, a concept for subdividing sex differences into three major types has been proposed (McCarthy et al., 2012). The first type assumes an absolute sexual dimorphism, the second type explains sex differences along a continuum, and most interestingly, the third type involves instances where the sexes either start on different levels and converge to the same end-point or start at the same level and diverge in response to an environmental challenge. In line with the third type are recent findings in degus using an early life stress paradigm (Kunzler et al., 2013), which revealed that both unstressed sexes displayed comparable levels of catecholaminergic fiber density in the orbitofrontal cortex. This pattern diverged in response to ELS exposure with the males ending up with elevated fiber densities. In contrast, the medial prefrontal cortex of unstressed male controls displayed denser catecholaminergic fibers than control females, which decreased after ELS exposure down to the level of female controls. Several other studies revealed that early life adversity reduces pre-existing differences between males and females, which might be interpreted as feminization or masculinization of behavior, brain structure, physiology and gene expression (Reznikov et al., 1999; Bowman et al., 2004; Gue et al., 2004; Mandyam et al., 2008; Zuena et al., 2008; Biala et al., 2011; Bock et al., 2011; Salomon et al., 2011).

## Conclusion

It is obvious that there are a high number of inter individual differences in the response and outcomes of early life stress. This indicates that predispositions exist, defining an individual's susceptibility or resilience against adverse environmental influences. In this context Nederhof and Schmidt (2012) use the term programming sensitivity defined as the ability of an individual to adapt its phenotype in response to environmental cues to increase its fitness under similar environmental conditions in the future. In light of the mismatch stress hypothesis (Oitzl et al., 2010; Schmidt, 2011; Frankenhuis and Del Giudice, 2012) this may explain why some individuals as adults benefit from their programming sensitivity during early life, even if they experience adverse environmental influences (match), while others experiencing the same situations may end up with disease-like symptoms because of a different programming sensitivity (Nederhof and Schmidt, 2012) and/or genetic predisposition. Predispositions are also an important aspect in the two- and three-hit concepts of vulnerability and resilience to stress-related mental disorders, which are related to the cumulative stress hypothesis stating that in a given context vulnerability is enhanced when failure to cope with adversity accumulates (McEwen, 1998; Taylor, 2010; Daskalakis et al., 2013). The three hit concept is based on gene-environment interactions during critical phases of perinatal and juvenile brain development and defines the three hits as follows: hit-1: genetic predisposition, hit-2: early-life environment, and hit-3: later-life environment (Daskalakis et al., 2013).

**Figure 3:**
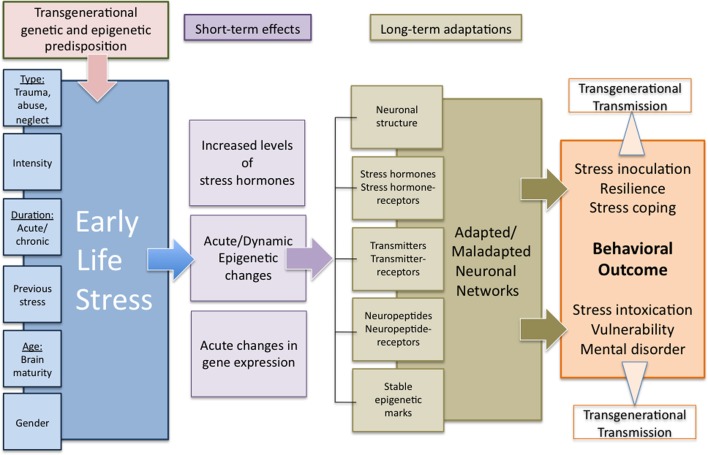
**Transgenerational programming of neuronal networks by early life stress**. The extent to which early life stress results in either an adaptive or a maladaptive behavioral outcome depends on a variety of environmental and internal influences and includes transgenerational components in terms of both genetic predisposition as well as acquired and transgenerationally transmitted epigenetic marks. Lasting behavioral changes are supposed to derive from acute and dynamic stress-induced alterations in gene expression as well as endocrine and epigenetic changes in interaction with the particular environmental and internal conditions resulting in stable long-term changes in neuronal networks. The adaptive or maladaptive changes determine the particular behavioral outcome under normal or stressful conditions in a positive or negative way. Additionally, these multi-level long-term adaptations might be transferred to following generations by epigenetic or behavioral mechanisms, representing a transgenerational predisposition for the adaptation to early life experiences.

Here we expand these concepts specifically by incorporating genetic predisposition and programming sensitivity to the context of experience-expectancy and transgenerational epigenetic traits (see Figure 3). Based on the above cited literature we propose the view that an individual predisposition of stress responsive peripheral and central nervous systems is not only a genetic predisposition (for example polymorphisms or gender) but acts in close cooperation with epigenetic predispositions. These epigenetic traits are inherited and resemble the sum of transgenerational experiences (from parents, grandparents) defining a familial prediposition to adverse environmental challenges. The interplay between genetic and epigenetic predispositions provides the framework underlying experience expectancy. For the adaptive maturation of stress responsive neural and endocrine systems it is essential to be challenged by a certain amount and quality of adverse experience, thus, these systems expect and “wait” for sufficient environmental input to optimize their functions. Repeated environmental challenges can be viewed within the framework of the match/mismatch hypothesis, the outcome, psychopathology or resilience, depends on the respective predisposition and on the context later in life.

### Conflict of interest statement

The authors declare that the research was conducted in the absence of any commercial or financial relationships that could be construed as a potential conflict of interest.
